# Broadly neutralizing antibodies: What is needed to move from a rare event in HIV-1 infection to vaccine efficacy?

**DOI:** 10.1186/s12977-018-0433-2

**Published:** 2018-07-28

**Authors:** Harini Subbaraman, Merle Schanz, Alexandra Trkola

**Affiliations:** Institute of Medical Virology, University of Zurich, Zurich, Switzerland

## Abstract

The elicitation of broadly neutralizing antibodies (bnAbs) is considered crucial for an effective, preventive HIV-1 vaccine. Led by the discovery of a new generation of potent bnAbs, the field has significantly advanced over the past decade. There is a wealth of knowledge about the development of bnAbs in natural infection, their specificity, potency, breadth and function. Yet, devising immunogens and vaccination regimens that evoke bnAb responses has not been successful. Where are the roadblocks in their development? What can we learn from natural infection, where bnAb induction is possible but rare? Herein, we will reflect on key discoveries and discuss open questions that may bear crucial insights needed to move towards creating effective bnAb vaccines.

## Background

The potency of the neutralization response to HIV-1 has long been underappreciated, as most of the antibodies identified were type-specific or had only limited breadth [1]. This only changed in the early 2000s, with the discovery of the new classes of potent bnAbs [1, 2]. This positioned bnAb responses as the major goal of vaccine development and in prevention. Despite their potency and breadth, there is no evidence that bnAbs ameliorate disease progression in natural infection, as they are subject to viral escape like any autologous neutralization activity [3–6]. Application of bnAbs as a therapeutic vaccine in established infection is therefore limited to settings where activity over shorter intervals is required. This is similar to what has been discussed in treatment combinations that aim to eliminate the latent HIV-1 reservoir [7]. In prevention, where bnAbs are considered both as a passively administered drug and are intended to be elicited by vaccines, their potential is obvious and has been underscored by numerous animal studies [1, 2, 8]. The virus inoculum that needs to be combatted to prevent transmission is low [9]. bnAbs have a window of opportunity to prevent infection in the absence of an established cellular HIV reservoir and potentially in concert with effector functions of the immune system. However, challenges for the use of bnAbs for prevention remain high. Therapeutically, bnAbs could be selectively applied to patients harboring sensitive strains. In contrast, antibodies elicited by a vaccine and/or used in a prevention setting must be highly potent and have exceptional breadth, targeting a wide spectrum of globally circulating HIV-1 strains.

Discoveries in the last decade have revealed that such elite neutralizing responses occur during natural infection, but are rare [10–14]. Initial hopes that delineation of the epitopes of identified elite bnAbs would allow rapid construction of matching immunogens were not fulfilled. Reverse vaccinology and structure-guided immunogen design based on these elite bnAbs brought much momentum to the field. The most recently developed immunogens are the first that induce Tier-2 neutralizing activity, but none of them has evoked a bnAb response to date [15, 16]. Therefore, natural infection remains the only system in which we can decipher the parameters that drive the evolution of bnAbs. Herein, we review key observations made on the determinants of bnAb development by studying both individual bnAb donors and HIV-1 patient cohorts and mark open questions that need to be addressed.

## The complex interrelation of bnAb maturation and virus escape

Focused efforts to decipher antibody maturation pathways alongside virus evolution have facilitated reconstruction of bnAb development in some individuals [3, 4, 17–23]. It is generally appreciated that a complex interplay between escape virus and Ab response is needed to trigger bnAb evolution [22]. The role of virus diversification, including superinfection, which can precede the emergence of breadth, has been underscored in several studies [3, 5, 20, 22, 24–27]. Whether virus diversification is driving or is, in fact, a consequence of bnAb development remains difficult to dissect, as Env variability also increases in response to bnAb pressure [28–31]. This iterative and circular nature of virus and antibody co-evolution that occurs in natural infection will be difficult to mimic by vaccination. Deciphering cause and consequence, as well as defining minimal necessary components of bnAb development will be key for designing successful vaccine regimens.

Prolonged bnAb maturation does not necessarily lead to improved breadth. The highest frequency of bnAb activity is observed after approximately 3 years of infection [12, 14, 32–34]. However, further prolonged replication does not increase frequencies [12, 14, 32, 33] and can even lead to a decrease or loss of bnAb activity [20]. Hence, while a relatively long exposure to viral antigen is commonly needed in adult HIV-1 infection to mount bnAb responses, continued adaptation of early bnAbs can also lead to “off-track” antibodies that lack breadth but have increased autologous strain specificity [3, 17, 22, 35]. Likewise, continued somatic hypermutation (SHM) will always generate “dead-end” antibodies harboring mutations that impede further development of functional antibodies [3, 17, 36]. SHM observed in isolated bnAbs often includes mutations not required for breadth development [37]. Short bnAb maturation phases with targeted breadth evolution must therefore be an ultimate goal for vaccine design.

Virus escape creates new epitopes that allow bnAb responses to mature [5, 24]. Slow virus escape may be beneficial for bnAb development, as this prolongs exposure to the bnAb-sensitive epitope, thereby extending antigenic stimulation and increasing chances of bnAb maturation [38–40]. Along these lines, partial evasion of virus from bnAbs, as can occur during cell–cell transmission [38, 41–43], allows the bnAb-sensitive virus to persist, ensuring sustained epitope presentation to the maturing antibody.

The virus population is not only shaped by the bnAb lineage, but is also subject to pressure of the vigorous type-specific antibody response each patient mounts. This can, in turn, substantially impact bnAb evolution. Early bnAb development may be compromised through competitive exclusion by strain-specific antibodies that target the same epitope [44, 45]. Yet, cooperation between different antibody lineages may also facilitate bnAb development by driving the virus into escape mutations that prevent full escape from bnAb lineages [18, 19, 40]. Antibody helper lineages can be strictly strain-specific [40] or mature into bnAbs [19]. Understanding whether the development of multiple bnAb lineages [5] and bnAb/helper [18] tandems are common or rare events will be essential to appreciate (1) their importance and (2) the need to incorporate similar help into vaccination strategies. Of note, recent reports have suggested that passively administered bnAbs may perform antibody helper functions. Therapeutic administration of the bnAb 3BNC117 to viremic individuals enhanced heterologous plasma neutralization breadth beyond escape to 3BNC117 [46]. Passive immunization of newborn macaques with nAbs at sub-neutralizing doses before oral SHIV challenge led to rapid elicitation of an autologous neutralization response and early control of viremia [47].

## Factors that drive bnAb development

**Fig. 1:**
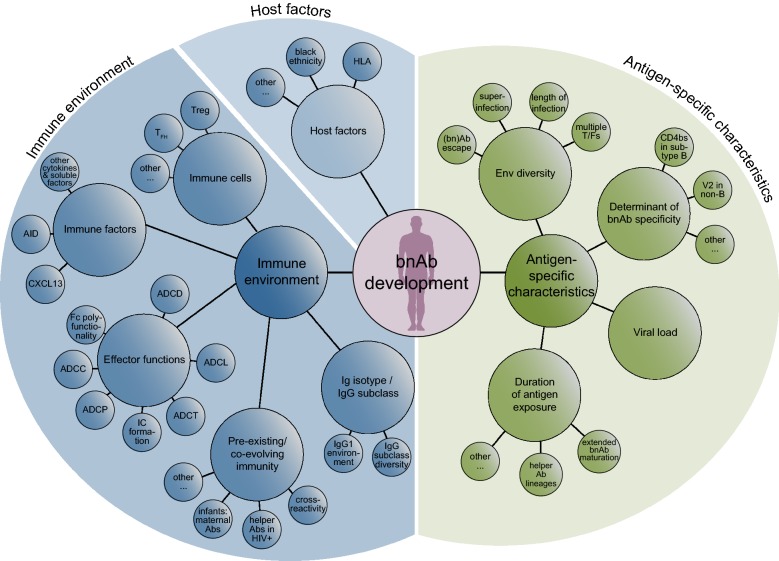
Parameters implicated in bnAb development

A range of factors that promote bnAb development has been implicated and several confirmed across different cohorts [6, 11, 12, 14, 32, 33, 48–50] (Tables 1, 2 and Fig. 1). These studies highlight that a combination of partly interdependent factors, which are linked with disease progression, direct bnAb development. Several prominent drivers of bnAb development are linked to persistent antigenic stimulation. The independent impact of viral load, infection length and diversity on bnAb frequency has been demonstrated [12, 14, 33, 48, 49]. These factors act in concert to ensure consistent antigenic stimulation. High viral load was the factor most consistently found to positively influence neutralization breadth across cohorts [6, 11, 12, 14, 32, 33, 48–51]. However, while rates of bnAb activity are significantly higher amongst individuals with high viral load, breadth can also develop in HIV-1 controllers, although commonly at lower frequency [11, 34, 39, 51–53]. Thus, high antigen loads promote bnAb evolution but are certainly not the sole driving force.

**Table 1:** Overview of patient cohort studies investigating viral and disease factors linked with bnAb development

Reference	Number of subjects^a^	Investigated viral and disease factors	Association with breadth	Additional information on investigated parameter
Doria-Rose et al. [11]	103	*Viral load*	*Positive*	Contemporaneous
		Infection duration	None	
		Transmission mode	None	
		CD4+ T cell levels	None	Contemporaneous
		History of ART use	None	
Euler et al. [6]	82	*Viral load*	*Positive*	Set point
		*CD4+ T cell levels*	*Negative*	Set point
		Disease progression	None	
Gray et al. [33]	40	*Viral load*	*Positive*	Set point
		*CD4+ T cell levels*	*Negative*	6 months post-infection
		*CD4+ T cell decline*	*Positive*	Difference between levels pre-infection and at 6 months
Landais et al. [14]	439	*Viral load*	*Positive*	Set point
		*Infecting subtype*	*Positive*	Subtype C infection
		*Infection duration*	*Positive*	
		Transmission mode	None	
		*CD4+ T cell levels*	*Negative*	All tested time points beyond 6 months post-infection
Mikkell et al. [32]	38	*Viral load*	*Positive*	Contemporaneous
Piantadosi et al. [48]	70	*Viral load*	*Positive*	Set point
		*Viral diversity*	*Positive*	Env
		CD4+ T cell levels	None	Contemporaneous
		Disease progression	None	
Rusert et al. [12]	4484	*Viral load*	*Positive*	Contemporaneous
		*Viral diversity*	*Positive*	Pol
		Infecting subtype	None	
		*Infection duration*	*Positive*	
		*Transmission mode*	*Weakly positive*	Modestly higher bnAb activity in IDUs
		*CD4+ T cell levels*	*Weakly positive*	Contemporaneous; modest association with cross-neutralization activity
Sather et al. [49]	39	*Viral load*	*Positive*	Average of all tested time points
		*Infection duration*	*Positive*	
		CD4+ T cell levels	None	Average of all tested time points
van Gils et al. [50]	35	*Viral load*	*Positive*	Set point
		*CD4+ T cell levels*	*Negative*	Set point

^a^Indicates total number of subjects included. Specific analyses may have been carried out on subsets of these numbers

**Table 2:** Overview of patient cohort studies investigating host and immune factors linked with bnAb development

Reference	Number of subjects^a^	Investigated host and immune factors	Association with breadth
Boliar et al. [90]	41	*Total plasma IgG*	*Positive*
		B cell expression of:	
		PD-1	None
		BTLA	None
		Ki67	None
		CD95	None
Cohen et al. [78]	15	*CXCR5* ^*+*^ *CD4* ^*+*^ *T cells*	*Positive*
		*CXCR5* ^*+*^ *PD-1* ^*+*^ *CD4* ^*+*^ *T cells*	*Positive*
		*CXCR5* ^*+*^ *PD-1* ^*+*^ *ICOS* ^*+*^ *CD4* ^*+*^ *T cells*	*Positive*
		*Plasma CXCL13*	*Positive*
		Plasma IL21	None
		Plasma BAFF	None
		Other cytokines and chemokines	None
		CD3^−^CD19^+^ CD27^−^ naïve B cells	None
		CD3^−^ CD19^+^ CD27^+^ memory B cells	None
		Env-specific CD3^−^ CD19^+^ CD27^+^ gp120^+^ memory B cells	None
		CXCR5 expression on B cells	None
		*Expression of activation-associated genes*	*Positive*
		*Expression of IFN-stimulated genes* *IFI27* *and* *ISG15*	*Positive*
		Expression of *CXCL13* and *RGS13*	*Positive*
Doria-Rose et al. [51]	148	Total CD19^+^B cells	None
		CD19^+^IgG^+^ B cells	None
		CD19^+^ CD27^+^ memory B cells	None
		CD19^+^ CD20^−^ CD27^+++^ CD38^+++^ plasmablasts	None
		Env-specific CD19^+^ gp140^+^ B cells	None
Doria-Rose et al. [11]	103	Ethnicity	None
		Gender	None
		Age	None
		HLA genotype	None
Dugast et al. [53]	163	*Plasma CXCL13*	*Positive*
		*Plasma sCD40L*	*Positive*
		*Plasma RANTES*	*Positive*
		*Plasma TNF-α*	*Positive*
		*Plasma IP-10*	*Positive*
		Other cytokines and chemokines	None
Havenar-Daughton et al. [81]	228	*Plasma CXCL13*	*Positive*
Kadelka et al. [54]	4281	*IgG1, IgG2 and IgG3 binding to trimeric Env*	*Positive*
		*IgG1 binding to Env-gp120*	*Positive*
		*IgG2 binding to Env-gp120*	*Positive*
		*IgG3 binding to MPER*	*Positive*
		*IgG3 binding to p17 and p24*	*Positive*
Landais et al. [14]	439	Gender	None
		Age	None
		Geographical origin	None
		*Total plasma IgG*	*Positive*
		*Env-specific IgG binding titer*	*Positive*
		Env-specific IgG binding avidity	None
		*HLA genotype*	Positive (HLA-A*03)
		KIR genotype	None
Locci et al. [79]	328	CXCR5^+^CD4^+^ T cells	None
		ICOS^+^PD-1^+++^ CXCR5^+^ CD4^+^ T cells	None
		*PD-1* ^*+*^ *CXCR3* ^*−*^ *CXCR5* ^*+*^ *CD4* ^*+*^ *memory Tfh cells*	*Positive*
		CXCR3^−^ CXCR5^+^CD4^+^ T cells	None
		PD-1^+^CXCR3^+^ memory Tfh cells	None
Mabuka et al. [83]	22	*Plasma CXCL13*	*Positive*
		Plasma BAFF	None
		CD19^+^CD21^−^CD27^+^ activated memory B cells	None
		CD19^+^CD21^−^CD27^−^ tissue-like memory B cells	None
		CD19^+^CD21^+^CD27^+^ resting memory B cells	None
		CD19^+^CD27^+^CD38^+++^ plasmablasts	None
Mikkell et al. [32]	38	CD4+ and CD8 + T cell expression of:	
		Ki67	None
		CD57	None
		*CD38*	*Positive (CD4+ T cells)*
		*PD-1*	*Positive (CD4+ T cells)*
		HLADR	None
Moody et al. [75]	239	HLA genotype	None
		*Plasma autoantibodies*	*Positive*
		*PD1* ^*+*^ *CXCR3* ^*−*^ *CXCR5* ^*+*^ *CD4* ^*+*^ *resting memory Tfh cells*	*Positive*
		*CD25* ^*+*^ *Foxp3* ^*+*^ *CD4* ^*+*^ *Treg cells*	*Negative*
		CD25^+^Foxp3^+^CXCR5^+^CD4^+^ follicular Treg cells	None
		*PD-1 expression on CD25* ^*+*^ *Foxp3* ^*+*^ *CD4* ^*+*^ *Treg cells*	*Positive*
		*PD-1 expression on CD25* ^*+*^ *Foxp3* ^*+*^ *CXCR5* ^*+*^ *CD4* ^*+*^ *follicular Treg cells*	*Positive*
		*HLA-DR expression on CD4* ^*+*^ *Treg cells*	*Positive*
		*CTLA-4 expression on CD4* ^*+*^ *Treg cells*	*Positive*
		*LAG-3 expression on CD4* ^*+*^ *Treg cells*	*Positive*
		Genome-wide mutations	None
Ranasinghe et al. [77]	67	*Gag-specific CD4* ^*+*^ *responses*	Positive
		*Gp41- specific CD4* ^*+*^ *responses*	Positive
		Gp120-specific CD4^+^ responses	None
Richardson et al. [82]	23	ADCC	None
		ADCP	None
		*ADCD*	*Positive*
		*ADCT*	*Positive*
		*Fc polyfunctionality*	*Positive*
		*FcR binding*	*Positive*
		*C1q binding*	*Positive*
		*IgG subclass diversity*	*Positive*
		*IgG2 binding to trimeric Env, gp120, V3*	*Positive*
		*IgG2 binding to p24*	*Positive*
		*IgG4 binding to trimeric Env, gp41, V2*	*Positive*
		*Plasma CXCL13*	*Positive*
		*AID expression in B cells*	*Positive*
Rusert et al. [12]	4484	*Ethnicity*	*Positive (blacks compared to whites)*
		*Gender*	*Weakly positive (modestly higher breadth in men)*
Sather et al. [49]	39	Env-specific IgG binding titer	None
		*Env-specific IgG binding avidity*	*Positive*
		CD8^+^ T cell levels	None

^a^Indicates total number of subjects included. Specific analyses may have been carried out on subsets of these numbers

Two other parameters linked to extended antigenic stimulation, virus diversity and length of infection, also increase the likelihood of bnAb elicitation [11, 12, 14, 48, 49]. This highlights that exposure to antigen over prolonged periods of time aids bnAb evolution. Env diversification inevitably increases during protracted infection, which is mostly driven by the consecutive rounds of Ab maturation and virus escape. Env diversity must therefore be viewed as both cause and consequence of neutralizing antibody maturation. Emerging escape variants may generate new epitopes on Env that engage novel antibody germlines and start a bnAb lineage, as demonstrated for individual donors [5, 24]. The presence of multiple transmitted founder (T/F) viruses as reported for intravenous drug users (IDUs), as well as in superinfection, similarly expose the immune system to high Env diversity. In a recent large cohort study, IDUs showed modestly higher frequency of bnAb activity [12, 54]. Whether this is a result of higher diversity or other factors intrinsic to HIV infection of IDUs remains to be determined. Env diversity has been postulated as a driver of bnAb activity in cases of superinfection [3, 20, 25–27]. Nevertheless, superinfection does not guarantee the development of bnAb activity, as recent studies revealed [55, 56]. Diversity may have a dual role in bnAb development. Highly diverse Env populations may have an increased chance to harbor a specific Env variant that is capable of initiating a bnAb lineage. Continuously increasing Env diversity, on the other hand, provides a means to support bnAb maturation by presenting multiple antigenic variants.

## The impact of the infecting virus

As evidenced by the unresolved impact of viral diversity in steering bnAb responses, the overall role of the infecting virus remains to be defined. What are the genetic determinants of the virus that trigger a bnAb lineage? As discussed above, it is possible that viral diversity can foster bnAb evolution, but whether diversity at the level of the infecting virus (superinfection, multiple T/F viruses) is decisive or not, has not been resolved.

Understanding the role of the T/F viruses may be key [9]. While no large differences in bnAb elicitation have been seen in male to male and heterosexual transmission [11, 12, 14], bnAb activity is found more frequently after mother to child transmission [57, 58] (see section below) and possibly also in IDUs [12]. Whether the transmitted virus or other factors that differ in these settings underlie the elevated chances to develop bnAb responses needs to be dissected. Differential glycosylation, variable loop length and specific motifs linked with certain bnAb specificities may play a role, but could also differ dependent on subtype and transmission mode [59–64]. However, causal relationships between prospective Env features and neutralization breadth remain difficult to establish. This is also true for deciphering Env determinants that trigger germline precursors of bnAb lineages [65–69]. With few exceptions [19, 20, 22], inferred germline versions of bnAbs often lack measurable binding activity to Env probes, which are efficiently recognized by the mature bnAbs [65–67, 69–71]. This suggests that Envs with distinct characteristics are needed for triggering these bnAbs. Intriguingly, some reports suggest that inferred germline ancestors of Abs with non/low neutralizing activity frequently bind a range of recombinant Envs with high affinity [45, 72]. Larger numbers of bnAbs and non-neutralizing antibodies need to be investigated to confirm this disparity. However, based on their comparative ease to engage diverse Env variants, non/low-neutralizing antibodies may have a selection advantage in the germinal center (GC) reaction.

A main issue that has not been clarified is whether the capacity of a virus to induce a bnAb is (1) restricted to a specific Env variant that is only transiently present and lost upon Env evolution or (2) preserved over prolonged time periods and may even be stable over multiple transmissions. Despite continuous Env evolution, there is emerging proof that some Env traits are stable and evoke similar responses. Clear evidence for this stems from the comparison of bnAb specificities mounted in subtype B infection compared to non-B infection. Non-B infection proved superior in mounting V2-glycan-directed responses [12], which is supported by the fact that none of the isolated potent V2 bnAbs stems from subtype B infection or targets subtype B viruses efficiently [73]. In contrast, subtype B viruses were more effective in mounting CD4bs responses [12]. Thus, while the genetic subtype had no impact on the frequency of bnAb activity, it shaped the specificity of the response. Thus, specific Env features in the respective virus subtypes that direct the immune response towards a certain specificity must exist.

In principle, bnAb development could be facilitated by a range of phenotypic virus features that influence epitope exposure and accessibility. These include, Env conformation and stability, the degree of shielding, and Env density on the virion surface. Unravelling genetic and/or structural Env features that promote or suppress bnAb development will be crucial for vaccine success.

## Immune environment

bnAbs often show poly/autoreactivity (reviewed in [74]) and autoantibody frequency has been reported to be high in bnAb-developing individuals [75]. Autoreactivity is particularly strong in the case of MPER bnAbs 2F5 and 4E10, and immune tolerance mechanisms must be overcome for the induction of MPER bnAb responses in humanized mouse models [74]. Therfore, reduced immune control may foster bnAb development in certain cases. An indication that this may occur in natural HIV infection stems from the observation that low CD4^+^ T cell levels and a high rate of CD4^+^ T cell decline appear to be linked with broad neutralization in some cohorts [6, 14, 33, 50]. While the distribution of CD4^+^ T cell subsets was not assessed directly in most studies, lower CD4^+^ T cell levels may implicate reduced levels of regulatory CD4^+^ T cells that may feed into bnAb development. However, a link between low CD4^+^ T cell levels and neutralization breadth was not observed universally [11, 48, 49, 51]. This may in part be due to the inverse association between CD4^+^ T cell counts and viral load [76]. With viral load confirmed as an independent determinant of bnAb evolution, assessing the specific effect of CD4^+^ T cell levels requires cohorts with the power to dissect confounding factors. Controlling for a range of factors, including viral load and infection length, the Swiss 4.5k Screen studying 4484 individuals found only a marginal independent impact of lower CD4^+^ T cell levels in individuals that mounted low levels of breadth and not those who had potent bnAb activity [12]. Of note, in the same cohort CD4^+^ T cell levels were found not to correlate with binding antibody responses to gp120, but a strong inverse effect on MPER IgG1 levels was observed [54]. This supports the notion that while MPER antibodies may benefit from a partially impaired immune system, the majority of bnAbs will not benefit from an environment with reduced CD4^+^ T cells. In fact, a recent study suggests that perturbations in the CD4^+^ T cell environment are linked with neutralization breadth. bnAb inducers had lower numbers of PD-1^high^ regulatory CD4^+^ T cells, highlighting a decreased regulatory capacity [75]. Extensive levels of SHM are commonly found in bnAbs. HIV-specific CD4 T cell responses have been linked with neutralization breadth [77] and elevated GC activity in bnAb inducers has been indicated by increased frequency of circulating memory T follicular helper (T_FH_) CD4^+^ cells, particularly early in infection [75, 78–80]. Likewise, elevated plasma levels of CXCL13, a cytokine involved in B cell migration to the GC, and increased expression of activation-induced cytidine deaminase (AID), the enzyme that orchestrates Ig hypermutation and class switch recombination, was observed in bnAb-inducers [53, 78, 81–83].

What stimulates such beneficial immune environments has not been resolved, but host genetics are implicated by several lines of evidence. A decreased prevalence of the protective allele HLA-B*57 [84] (linked with slower disease progression) and expression of a specific HLA allele (HLA-A*03) [14] have been implicated. Likewise, other HLA variants and SNPs within the MHC complex may directly or indirectly impact bnAb evolution via influencing viral load [11, 14, 75, 84]. As highlighted by CD4bs bnAbs, some bnAb specificities are restricted to a limited set of Ig heavy chain germline alleles, which encode signature features relevant for the specific epitope recognition [71]. Hence, it is possible that the ability to produce these types of antibodies is genetically restricted. However, no overall difference in the immunoglobulin gene repertoires of bnAb inducers and non-neutralizers has been observed to date [85].

In support of a strong genetic influence, individuals with black ethnicity were found to more frequently induce bnAb activity compared to white study participants [12] and have enhanced antibody binding IgG1 responses [54]. Ethnicity-dependent differences in the antibody response to a HIV-1 gp120 immunogen have independently been reported [86]. While the influence of socio-economic factors cannot be excluded, focused studies to reveal a potential genetic determinant are highly warranted. Depending on the genetic determinants identified this may or may not be relevant to vaccines. Nevertheless, their contribution needs to be determined to understand if future HIV bnAb-inducing vaccines can be expected to be effective in the whole population or only in proportions thereof.

## Specific antibody signatures are linked with neutralization breadth

With increased knowledge about parameters that are linked with bnAb development in natural infection (Tables 1, 2 and Fig. 1), opportunities to define factors that promote bnAb activity and surrogate markers that predict bnAb evolution are now within reach. An important step towards this came from recent systems serology studies that investigated multiple aspects of the antibody response repertoire in vaccine recipients, non-neutralizers and individuals who developed broad neutralization activity [53, 54, 82, 87–89].

Titer [14] and avidity [49] of IgG binding responses to Env-based antigens have been reported to correlate with neutralization breadth. IgG subclass distribution of the HIV-1 antigen response shows a distinct IgG1-driven pattern in bnAb inducers, suggesting the presence of immune regulatory mechanisms that promote IgG1 responses [54]. In addition, elevated IgG2 and IgG4 responses against HIV antigens in bnAb-inducers compared to non-neutralizers have been observed early in infection [82]. This may in part reflect higher antigen exposure, as IgG2 anti-Env responses are strongly driven by viral load [54]. Diverse parameters that influence the development of neutralization breadth (Tables 1, 2) also impact binding antibody responses to HIV-1 but in an antigen-dependent manner [54], underscoring the complexity of these interrelations. Differential antibody profiles observed in response to HIV-1 Env vaccination regimens [87–89] suggest that modulating the immune response towards patterns that may favor bnAb evolution could be possible. However, this requires detailed knowledge of which antibody features are needed, as well as defined strategies to shift responses in the desired direction.

The importance of effector functions in the protective effect of neutralizing antibodies has been long recognized and both activity of non-neutralizing and neutralizing antibodies in the context of effector functions implicated [53, 91–96]. These include immune complex (IC) formation, antibody dependent complement deposition (ADCD) and lytic (ADCL) activity, antibody dependent cytotoxicity (ADCC), antibody dependent trogocytosis (ADCT) and antibody dependent phagocytosis (ADCP). Increased effector activity of antibodies that bind to Env-expressing infected cells with high affinity, like bnAbs, may deliver superior antiviral activity allowing elimination of infected cells—a goal of HIV-1 cure approaches [94–96].

IgG subtypes differ in their capacity to deliver effector functions and differential glycosylation of the Fc also influences the interaction with Fc receptors and the efficacy of the effector responses [97]. Considerable evidence suggests that steering immune responses towards certain Ig subclasses and/or Fc modifications needs to be evaluated. HIV-1 controllers display antibody subclass profiles that are skewed towards IgG1 or IgG3 responses with the ability to coordinate several effector functions including ADCC and ADCP that suppress viral replication [98–100]. Similarly, immune correlates of protection in the RV144 trial have been linked to antibody effector functions [101, 102]. Systems serology approaches revealed that polyfunctional Fc-effector profiles of anti-Env responses are a critical component of viral control in natural infection [98–100, 103] and distinguish responses to different vaccine regimens [87–89]. When and where Fc effector activity is important, whether improved signaling capacity through immune complex formation or activation of the cell killing mechanism is required, needs to be determined. Common immune determinants that regulate both Fab and Fc mediated activities may be involved, since Fc polyfunctionality and potency early in infection have been associated with the propensity to develop neutralization breadth [82]. Increased levels of ADCD and ADCT were observed early (but not later) in infection in individuals who developed bnAbs [82]. This stresses the complexity in dissecting cause and consequence also in this context. We need to decipher to what extent Fc effector functions actively foster bnAb development and/or develop in parallel (as they depend on the same immune factors) in order to appreciate the importance of stimulating Fc effector environments by vaccination.

## HIV-1 infected infants develop bnAbs more frequently, more rapidly and with less SHM

While most bnAbs have high levels of SHM and evolved after prolonged maturation pathways, this seems not to be needed in all settings. A proportion of adults develop bnAb activity comparatively rapidly [12, 20, 22, 32]. In infant HIV infection, bnAb evolution is generally fast and, with more than 70% of cases developing breadth, also substantially more frequent than in adults [57, 58]. Deciphering the underlying causes of slow and rapid bnAb evolution in adults and infants may open new potentials for vaccine design and immunization strategies. The first infant bnAb characterized in detail highlights the potential [104]. In line with a more direct developmental pathway in infants, the N332 glycan-dependent supersite-targeting bnAb BF520.1, isolated from a HIV-1 infected infant 1 year post-infection, has notably low levels of SHM (6.6% nt) and lacks heavy and light chain indels compared to adult V3 glycan region-targeting bnAbs [104]. If conditions that allow rapid development of low SHM bnAbs like BF520.1 prove to be transferrable to other settings this may open immense potential to create an effective bnAb vaccine in adults. Pinpointing the causative effects will however be challenging. A range of factors differ between adult and infant infection and several may feed into each other. While it seems surprising, the infant immune system appears to provide a better setting for bnAb development. In early life, B cell responses are partially restricted, the Ig germ-line repertoire is not fully developed and the co-stimulatory network is not yet fully functional, leading to compromised B cell responses with lower SHM and heterogeneity [105, 106]. IgG subclass distribution differs markedly in infants with IgG1 and IgG3 levels, the most relevant subclasses for neutralizing HIV responses [54], rising sooner to adult-like concentrations than IgG2 and IgG4 [106, 107].

While antibodies with lower SHM may prove to be common in infants, it is intriguing that bnAbs with low SHM have not been described in adults. Does the adult immune system not favor the development of low SHM bnAbs? A potential scenario could be that cross-reactive antibodies that bind to HIV Env exist in adults. Following the dogma of original antigenic sin [108], instead of priming a de novo antibody response, the evolution of the Env antibody response would be restricted to affinity maturation of a pre-existing cross-reactive antibody. This may limit the response and require more extensive SHM to reach neutralization breadth. Of note, cross-reactivity with unrelated proteins and/or host antigens have been described for several HIV bnAbs [23, 109–111].

Vertical transmission is distinct from all other HIV-1 transmissions, as maternal antibodies are transferred and remain present for prolonged time periods. The role of maternal Abs in influencing transmission risk is still debated [112–115]. Irrespective of their potential in preventing transmission, it is intriguing to speculate that maternal antibodies may function as helper antibodies [19, 40]. The presence of maternal Abs may allow immune-focusing that aids neutralizing antibody development, as described for passive immunization of infant macaques [47]. Influence of maternal antibodies on vaccine responses in infants has been reported [107] and may comprise a number of mechanisms including activation of the immune system through IC formation that aid bnAb evolution. Specific immune-focusing of the infant response could also be envisaged through binding of immunodominant epitopes by maternal antibodies, shielding these from access by infant BCRs and thereby directing the Ab response to other sites without competing for survival signals in the GC reaction. In the setting of HIV-1 infection, this immediately raises the question whether neutralizing or non-neutralizing antibodies or even combinations thereof would be needed to create effective immune-focusing [116].

High viral load—a strong influence on bnAb evolution in adults—is frequent in infant HIV-1 infection in the first 2 years of life [117, 118] and may also promote infant bnAb development as seen in adult HIV infection [11, 12, 14, 32, 33, 48–50]. Importantly, vertical transmission may also create bottlenecks that differ from sexual transmission, potentially favoring transmission of phenotypically distinct strains that favor bnAb triggering. Defining the phenotypic properties of T/F viruses from vertical transmission cases that developed Ab breadth is of high relevance. The best case scenario for vaccine design would be that specific Env properties of infant T/F viruses are the underlying cause of the frequent and rapid bnAb evolution in infant HIV infection. Of note, the T/F virus BG505, derived from an infant that developed a bnAb response [119] is the currently most thoroughly studied trimeric Env immunogen and focus of diverse immunization strategies [119, 120].

## Conclusion

**Fig. 2:**
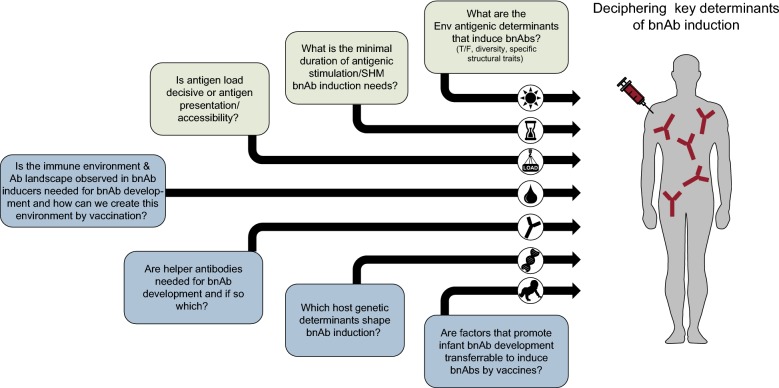
Key determinants of bnAb development that need to be deciphered

Detailed analysis of determinants that shape bnAb responses in natural infection have led to the identification of a range of factors that are linked with the development of neutralization breadth. However, so far we lack formal proof for which factors are ultimately causative in bnAb elicitation and which evolve alongside. Delineating the causes and consequences, as well as defining parameters that need to be incorporated into vaccine regimens will be critical. Figure 2 highlights key questions that need to be addressed, based on the current state of the field. Resolving these topics, ranging from the impact of the Env immunogen and the immune environment in which the bnAb response favorably evolves, to understanding why the infant HIV-1 infection is superior in mounting bnAb responses, will be challenging. However, this is ultimately necessary to move HIV vaccine design forward.
